# Inhibition of 3-Hydroxykynurenine Transaminase from *Aedes aegypti* and *Anopheles gambiae*: A Mosquito-Specific Target to Combat the Transmission of Arboviruses

**DOI:** 10.1021/acsbiomedchemau.2c00080

**Published:** 2023-02-16

**Authors:** Larissa G. Maciel, Matheus V. F. Ferraz, Andrew A. Oliveira, Roberto D. Lins, Janaína V. dos Anjos, Rafael V. C. Guido, Thereza A. Soares

**Affiliations:** † Department of Fundamental Chemistry, Federal University of Pernambuco, 50740-560 Recife, Brazil; ‡ Aggeu Magalhães Institute, Oswaldo Cruz Foundation, 50740-465 Recife, Brazil; § São Carlos Institute of Physics, University of São Paulo, 13563-120 São Carlos, Brazil; ∥ Department of Chemistry, University of São Paulo, 055508-090 Ribeirão Preto, Brazil; ⊥ Hylleraas Centre for Quantum Molecular Sciences, University of Oslo, 0315 Oslo, Norway

**Keywords:** noncompetitive inhibitor, metadynamics simulations, τRAMD, unbinding kinetics,, binding affinity, binding efficiency

## Abstract

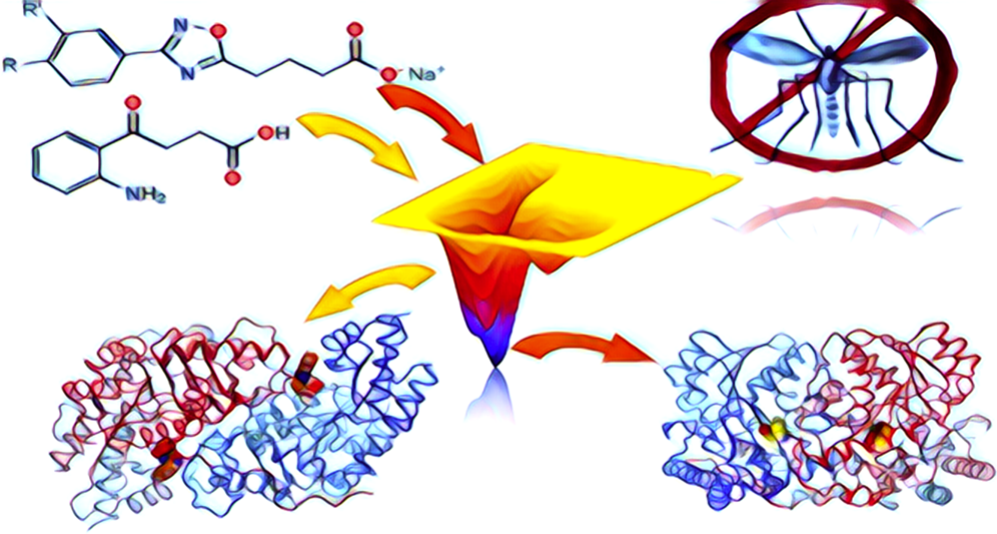

Arboviral infections such as Zika, chikungunya, dengue, and yellow fever pose significant health problems globally. The population at risk is expanding with the geographical distribution of the main transmission vector of these viruses, the *Aedes aegypti* mosquito. The global spreading of this mosquito is driven by human migration, urbanization, climate change, and the ecological plasticity of the species. Currently, there are no specific treatments for *Aedes*-borne infections. One strategy to combat different mosquito-borne arboviruses is to design molecules that can specifically inhibit a critical host protein. We obtained the crystal structure of 3-hydroxykynurenine transaminase (AeHKT) from *A. aegypti*, an essential detoxification enzyme of the tryptophan metabolism pathway. Since AeHKT is found exclusively in mosquitoes, it provides the ideal molecular target for the development of inhibitors. Therefore, we determined and compared the free binding energy of the inhibitors 4-(2-aminophenyl)-4-oxobutyric acid (4OB) and sodium 4-(3-phenyl-1,2,4-oxadiazol-5-yl)butanoate (OXA) to AeHKT and AgHKT from *Anopheles gambiae*, the only crystal structure of this enzyme previously known. The cocrystallized inhibitor 4OB binds to AgHKT with *K*_i_ of 300 μM. We showed that OXA binds to both AeHKT and AgHKT enzymes with binding energies 2-fold more favorable than the crystallographic inhibitor 4OB and displayed a 2-fold greater residence time τ upon binding to AeHKT than 4OB. These findings indicate that the 1,2,4-oxadiazole derivatives are inhibitors of the HKT enzyme not only from *A. aegypti* but also from *A. gambiae*.

## Introduction

The *Aedes aegypti* mosquito is the main transmission vector for several viruses, including the urban yellow fever (YFV), dengue (DENV), chikungunya (CHIKV), and Zika (ZIKV) viruses responsible for high rates of morbidity and mortality in tropical regions around the globe.^1^ Just for the dengue fever disease, it is estimated a total of 390 million virus infections per year worldwide (95% credible interval 284–528 million), of which 96 million (67–136 million) manifest clinically.^2^*A. aegypti* is closely associated with human habitation, thriving in densely populated regions. The female mosquito not only feeds on humans but also prefers to lay eggs in artificial water containers (e.g., water tanks, flower vases, pot plant bases, discarded tires) typically found around or inside houses.^3,4^ Furthermore, eggs can withstand desiccation for up to one year.^3^ Although *A. aegypti* is intolerant to temperate winters and its eggs are sensitive to frost, the current global climate changes may expand the geographical distribution of this disease vector.^5,6^ For instance, the European Centre for Disease Prevention and Disease Control considers that, with the increase on average temperatures, the coastal regions of the Mediterranean, Black Sea, Caspian Sea, and areas along large lowland rivers (Ebro, Garonne, Rhone, and Po) can become suitable habitats for *A. Aegypti*.^7^

As the effective immunization against arboviruses and all their many serotypes is not currently available,^1,8,9^ populational control of vector species becomes the most effective and affordable way to prevent disease transmission. This is particularly important because some arboviruses (e.g., DENV) are vertically transmitted in *Aedes* spp.^10−13^ It means that adult mosquitoes are infected through transovarial transmission and therefore do not have to feed in a viremic vertebrate host to infect a naïve host. This finding explains the persistence of the virus in nature even in the absence of viremic vertebrate hosts.^14^

A viable alternative to control the transmission of arboviruses by *A. aegypti* is the development of insect-specific chemical compounds capable of eliminating the larvae or the adult insect in water reservoirs within and around houses. As part of a decade-long effort, we have identified a class of 1,2,4-oxadiazole derivatives with larvicidal activity against the *A. aegypti* mosquito and low toxicity in mammals.^15−18^ Structural comparisons supported by computational calculations for the 1,2,4-oxadiazole nucleus against known inhibitors of different insect enzymes led to the identification of the enzyme 3-hydroxykynurenine transaminase (HKT) as a potential target for these larvicide prototypes with IC_50_ values ranging from 35 to 340 μM.^17,18^ HKT is a pyridoxal-5′-phosphate (PLP)-dependent enzyme responsible for the transamination of the toxic 3-hydroxykynurenine (3-HK) to the chemically stable xanthurenic acid (XA) in mosquito larvae and adults.^19^ This reaction is part of the main detoxification route regulating the degradation of tryptophan in mosquitoes.^20^ As in other PLP-dependent enzymes, the PLP cofactor in HKT acts as an electron sink.^21^ The accumulation of 3-HK in adult insects leads to the formation of reactive oxygen species with serious neuronal damage and organism death.^22,23^ The exogenous administration of 3-HK to insects also leads to irreversible paralysis and death.^23,24^ Besides being a mosquito-specific enzyme, HKT has less than 20% sequence identity with its human cognate kynurenine aminotransferases I (KAT-I) and II (KAT-II).^25,26^

These findings motivated the development of a high-yield procedure for the expression and purification of an active recombinant form of HKT from *A. aegypti* (AeHKT) to evaluate the inhibitory activity of the 1,2,4-oxadiazole derivatives.^17^ It demonstrated that this class of heterocyclic compounds are noncompetitive inhibitors of AeHKT, with the potential to disrupt the detoxification of the highly toxic 3-HK.^17^ However, the structure-based optimization of the inhibitory properties of 1,2,4-oxadiazoles has been precluded due to the unavailability of the experimental three-dimensional (3D) structure of AeHKT. Currently, the only X-ray structure of HKT available is from the malaria vector *Anopheles gambiae* (AgHKT) (PDB IDs: 2CH1 and 2CH2), which was cocrystallized with the competitive inhibitor 4-(2-aminophenyl)-4-oxobutyric acid (4OB).^27^ In fact, only 155 three-dimensional structures from mosquitoes were deposited in the Protein Data Bank until November 2022, attesting to the unexplored potential of insect proteins as drug targets for the control of vector-transmitted diseases. In this work, we solved the X-ray structure of AeHKT (PDB ID: 6MFB) to serve as a target for the discovery of new AeHKT inhibitors and optimization of 1,2,4-oxadiazole derivatives.^16−18^ We have further performed a series of metadynamics (Meta-MD) simulations to compute the multidimensional free energy surface (FES) for the binding process between the two HKT enzymes (AeHKT, AgHKT) and two inhibitors: 4OB, previously cocrystallized in complex with AgHKT,^27^ and a canonical representative of the 1,2,4-oxadiazole derivatives, sodium 4-(3-phenyl-1,2,4-oxadiazol-5-yl)butanoate (OXA).^17^ Since 4OB is the sole HKT inhibitor for which the enzyme-inhibitor complex crystallographic data is available, it serves as the gold standard for validation of new inhibitors via structure-based approaches. The reconstruction of the FES for the eight binding processes showed that OXA binds to both HKT enzymes with free energy values two-fold more favorable than the crystallographic inhibitor 4OB. These findings indicate that the X-ray structure of AeHKT is useful for structure-based drug design approaches and the 1,2,4-oxadiazole derivatives are prospective inhibitors of both AeHKT and AgHKT enzymes.

## Results and Discussion

### Overall X-ray Structure

**Figure 1:**
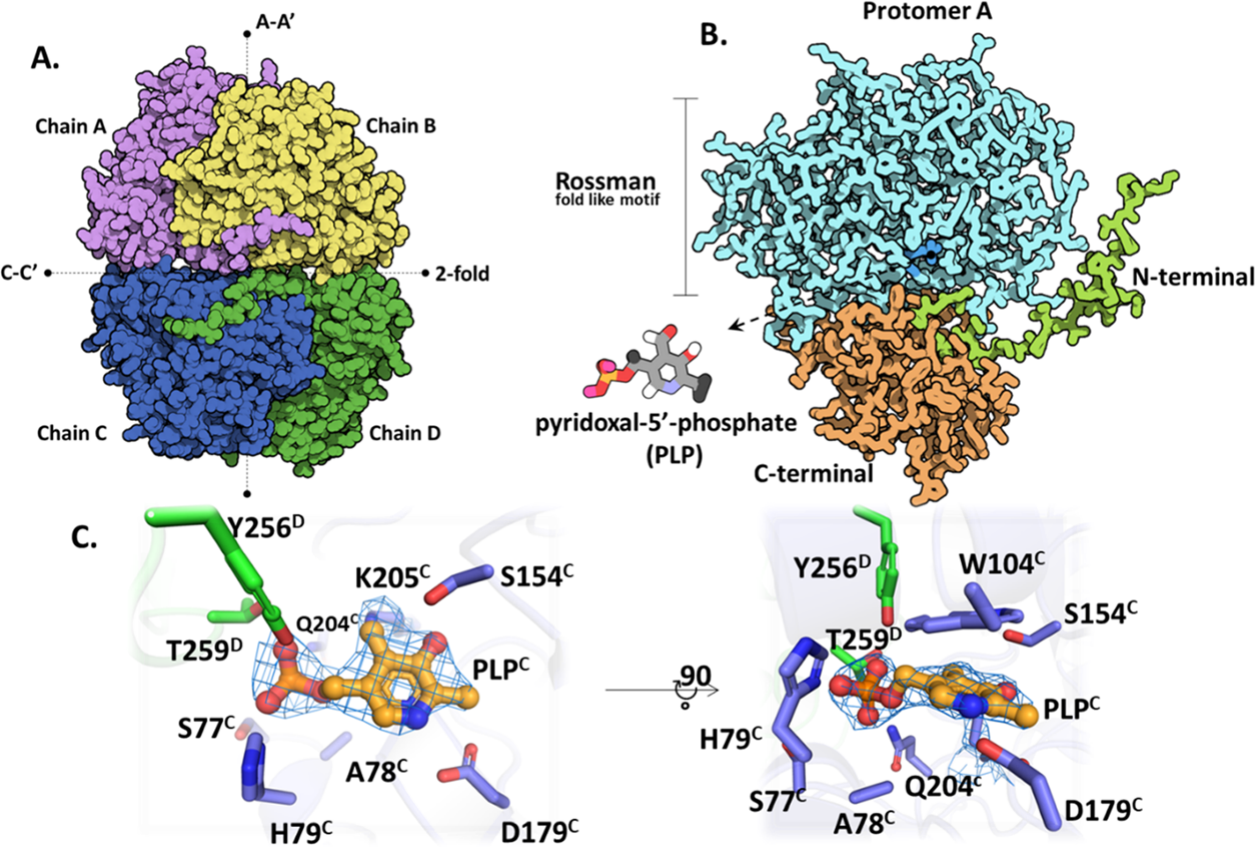
HKT overall structure. (A) The asymmetric unit of AeHKT is composed of four monomers (chain A in purple, chain B in yellow, chain C in blue, and chain D in green), related to A–A′ (homodimer) and C–C′ axes (asymmetric unit). (B) Detail of the protomer A fold, containing the N-terminal region in green, Rossmann-like motif in blue, and C-terminal in orange. The pyridoxal-5′-phosphate (PLP) cofactor is located at the intermediate region of the protomer, in which the active site is composed by the interface between two monomers of one homodimer. (C) Close-up of the active site at the homodimer interface (chain C in blue and chain D in green) and most important residues interacting with PLP (orange). PLP of chain C with an electronic density map is shown.

The AeHKT X-ray structure has four subunits (chains A to D), constituting two homodimers (AB and CD) in the asymmetric unit (Figure 1). Subunits A, B, and C had 385 out of 388 residues as expected according to the translated gene. Subunit D had 386 residues. The following residues A-Met^1^, A-Asp^386^, A-Tyr^387^, A-Val^388^, B-Asp^386^, B-Tyr^387^, B-Val^388^, C-Asp^386^, C-Tyr^387^, C-Val^388^, D-Tyr^387^, and D-Val^388^ were not modeled because of the absence of corresponding electronic density. The structure also showed four PLP molecules, one per monomer, and 370 water molecules. The overall fold of AeHKT is similar to that of other PLP-dependent enzymes, which share the same homology, type I. Important structural elements that hold the monomers together as a homodimer include the N-terminal arm, which connects the two domains at the enzyme’s N- and C-carboxyl terminus (Figure 1).

The protein architecture of the AeHKT enzyme matches the class of PLP-dependent type I aminotransferases, sharing several common features (e.g., the presence of the small, large, and N-terminal domains, the location of the active site between the monomeric units, the active enzyme being homodimeric).^28^ The small or C-terminal domain consists of residues 279–388 and shows a αβ complex motif containing antiparallel β-sheets pointed to the active site of the protein and protected by several α-helices. The large domain is built by residues 30–278 in a 3-layer αβα sandwich. This Rossmann-like motif consists of parallel β-sheets surrounded by α-helices on both sides, shielding the sheets from solvent. Finally, the N-terminal arm is an essential domain to keep the homodimers together (Figure 1). The latter displays a 2-fold symmetry axis, a common feature of type I aminotransferases.

### PLP Binding Site

Residues from both subunits contribute to the active site, which is found at the homodimer A–A′ contacts (Figure 1A). Considering that one PLP molecule is needed for each active site, there are four PLP molecules in the asymmetric unit. The PLP molecule binds to Lys205 (Schiff base) creating an inner aldimine via an imine reaction. Chains A and B, as well as chains C and D, were found to be part of two different homodimers in the solved structure of AeHKT at the C–C′ contact. The active site is positioned at the homodimer interfaces and composed of residues from both monomers. There is one PLP molecule per active site, resulting in four PLP molecules in the asymmetric unit. The PLP molecule is covalently bound to Lys^205^ through an imine reaction to form an internal aldimine (Schiff base) to form lysine-pyridoxal-5-phosphate (LLP). In the AeHKT solved structure, two homodimers are observed: one made of chains A and B, and another made of chains C and D. Since the homodimers are identical, the findings reported here for chains A and B are valid for chains C and D. The PLP binding mode is stabilized by π-interaction and van der Waals contacts with C-Trp^104^ and C-Val^181^ residues, respectively, and via a network of polar interactions with C-His^79^, C-Ser^154^, C-Asp^179^, C-Gln^204^, D-Tyr^256^, and D-Thr^259^ residues (Figure 1).

### Structural Comparison with Homologous Proteins

**Figure 2:**
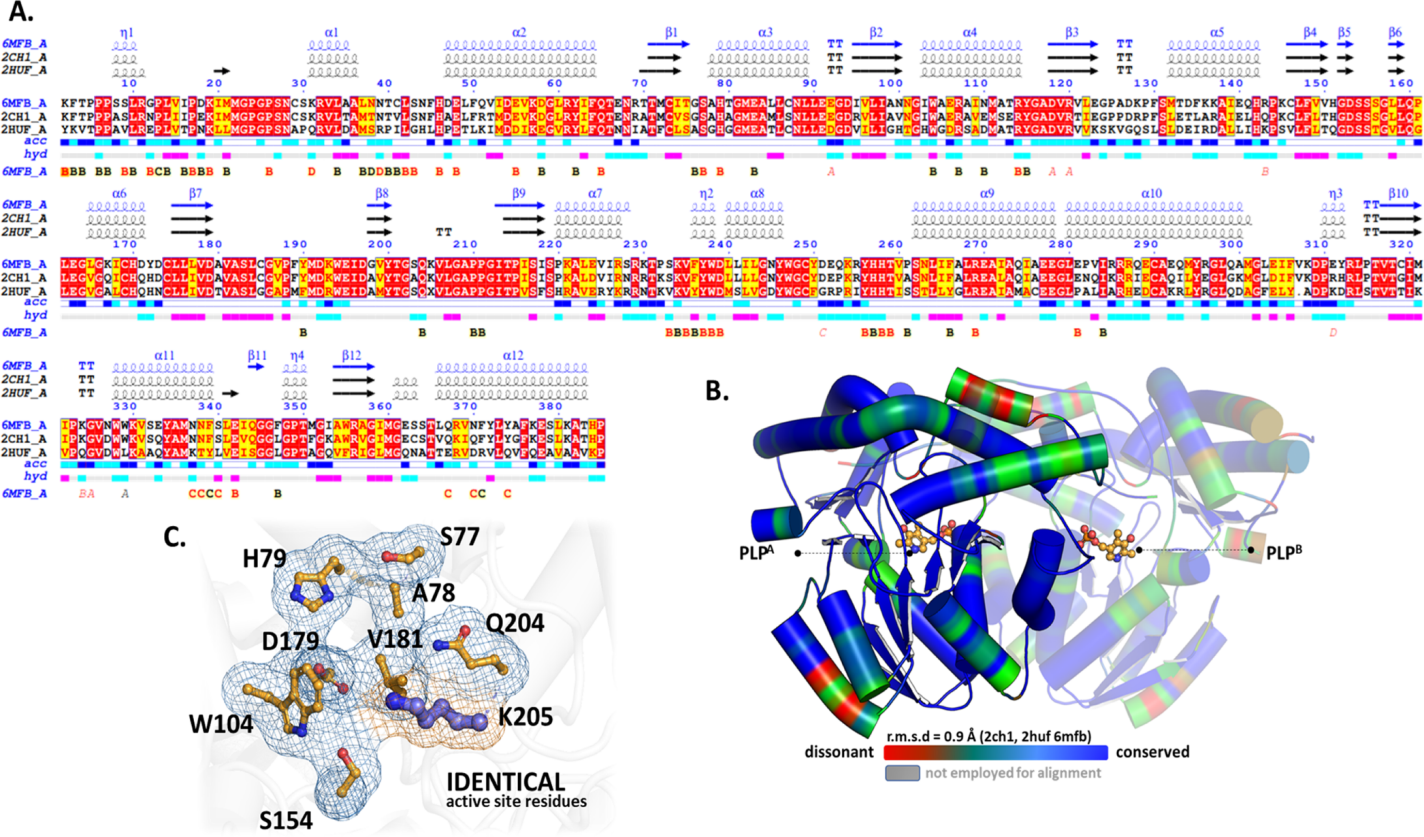
Comparison of AeHKT and homologues. (A) Sequence alignment for HKT from *A. aegypti* (AeHKT^6MFB^), *A. gambiae* (AgHKT^2CH1^), and AGT from *A. aegypti* (AeAGT^2HUF^). Alignment colors indicate red (conserved residues), yellow (conservative mutation), and white nonconservative mutations. Correspondent 6MFB secondary structures are indicated above the alignment using α (helix), ß (sheet), and η (turn). Evidence suggests that AeHKT/AgHKT are significantly closer together than AeAGT/AeHKT. (B) Structural superposition of AB homodimers of AeHKT, AgHKT, and AeAGT X-ray structures. Conserved and dissonant residues are highlighted in a ramp color between blue and red, respectively. Nonconservative mutations are seen in areas exposed to solvent (red). (C) Detail of the identical active site residues for superimposed X-ray structures of B (chain A). A common ancestor between AGT and HKT was duplicated to create HKT, as indicated by Chen et al.^29^ Consequently, the functional variations between 6MFB and 2HUF, where the only active site mismatch is between positions A78 and G79, seem to be due to substrate recognition rather than active site polymorphism. Alignment and a portion of the analysis had been completed with the ENDscript webserver.^31^

We performed structural and sequence alignments of AeHKT (PDB ID: 6MFB) with highly conserved tertiary structures in the family of PLP-dependent aminotransferases. Only two enzymes have sequence identity with AeHKT greater than 40%, namely, AgHKT (PDB ID: 2CH1) and AeAGT (PDB ID: 2HUF) (Figure 2). AeHKT shares sequence identities of 79% with AgHKT and 51% with AeAGT (Figure 2). The respective structures were superimposed with comparable root-mean-square deviation (RMSD) values of 0.4 and 0.8 Å, respectively, indicating a very similar folding. Although AeHKT and HsKAT II catalyze the conversion of 3-HK to XA, these enzymes share low sequence identity (below 20%),^17^ and the structural superposition of AeHKT (PDB ID: 6MFB) onto HsKAT I (PDB ID: 2VGZ) and HsKAT II (PDB ID: 2VGZ) yielded RMSD values greater than 10 Å. Furthermore, only AeHKT exhibits AGT activity, i.e., the ability to catalyze the transamination of alanine in the presence of glyoxylate as an amino group acceptor, indicative of evolutionary divergence.^29,30^ Therefore, the low sequence identity and dissimilar three-dimensional structure make the HsKATs an unlikely target for AeHKT inhibitors.

The PLP binding sites of AeHKT, AgHKT, and AeAGT share several highly conserved residues. Trp^104^ contributes to the π-stacking in the si face, while the Val^181^ residue interacts with PLP via hydrophobic interactions in the re face.^32^ The Ser^174^ and Asp^179^ residues play an important role in the PLP arrangement during catalysis.^32,33^ A-Ser^77^, A-His^79^, A-Gln^204^, B-Tyr^256^, and B-Thr^259^ residues are responsible for electrostatic interactions with the phosphate group of PLP (Figure 2).^27,34^ There are also nonconserved residues lining up the active site entry of different HKT enzymes, which are supposedly involved in substrate recognition. This is the case of Phe^347^/Leu^347^ and Met^351^/Phe^351^ residues present in AeHKT and AgHKT, respectively (Figure 2).^17^ The PLP binding site of AeHKT also shares evolutionarily conserved residues with AeAGT, namely, Trp^328^, Glu^342^, Gly^345^, Gly^346^, and Arg^356^. This is not surprising because AeHKT and AeAGT show the same AGT activity but in different mosquito life stages;^19^ HKT is upregulated in larvae stages and downregulated in pupae and adult ones. A comprehensive evolutive investigation of the HKT and AGT enzyme families supports a paralogous relation among these enzymes.^29^ HKT is only found in mosquitoes, being the only AGT homologue involved in the detoxification of 3-HK. Therefore, HKT likely originated from gene duplication of mosquito AGT in a common ancestral.^29^ Furthermore, mosquito-specific HKT is the only AGT homologue involved in detoxification of 3-HK, and it is only found in mosquitoes. The enzymes kynureninase and L-kynurenine aminotransferases are responsible for metabolizing 3-HK in other insects and mammals.^29^

## Computational Simulation

**Figure 3:**
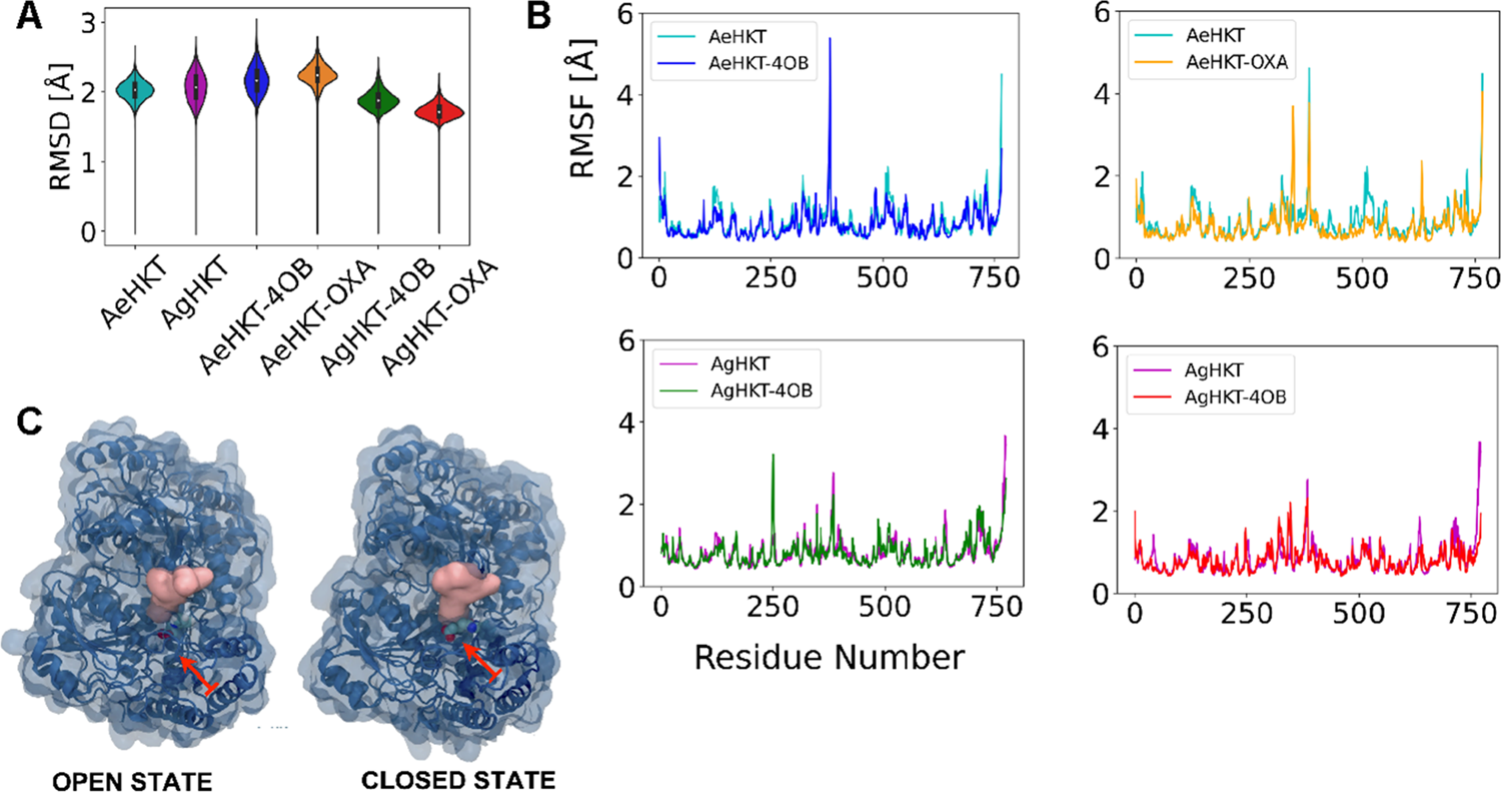
Time-series properties and conformations obtained from MD simulations. (A) Violin plot of average RMSD between the α carbons from the simulated structural ensemble and the crystallographic structure or lowest energy docking structure. (B) RMSF per residue of the α carbons calculated for the last 50 ns of simulation. (C) Open and closed conformations of AeHKT-OXA upon OXA binding. The residues A-Gln^253^-A-Arg^255^ in the loop connecting helices α8 and α9 are shown as solid pink surfaces. The OXA ligand is shown in van der Waals radii in element-color code. Red arrows show the position of the active-site embedded ligands.

Classical atomistic simulations were carried out to investigate the binding modes of the 4OB and OXA ligands to the AeHKT and AgHKT enzymes at the molecular level. Structural average properties derived from the simulations were recorded as a time series. The MD simulations indicated that AeHKT and AgHKT exhibit similar structural dynamics in the absence or presence of the ligands (Figure 3). The simulated structural ensembles showed root-mean-square deviation (RMSD) values of α-carbon atoms in the corresponding X-ray structures of 2 Å or lower (Figure 3A). These lower RMSD values suggest a considerable structural rigidity for the two enzymes without any significant conformational change. The structural rigidity of AeHKT and AgHKT can also be inferred from the root-mean-square fluctuation (RMSF) values, which probes the atomic displacements before and after ligand binding to AeHKT and AgHKT (Figure 3B). Hence, there was a common RMSF pattern for the free and ligand-bound enzymes, except for AeHKT-OXA. For this complex, there is an increase of RMSF values for A-Gln^253^-A-Arg^255^ residues, which was due to the opening and closing of the loop between helices α8 and α9 at the entrance of the binding site (Figure 3C). This behavior finding was not observed for the remaining systems, AeHKT-4OB, AgHKT-OXA, and AgHKT-4OB.

**Figure 4:**
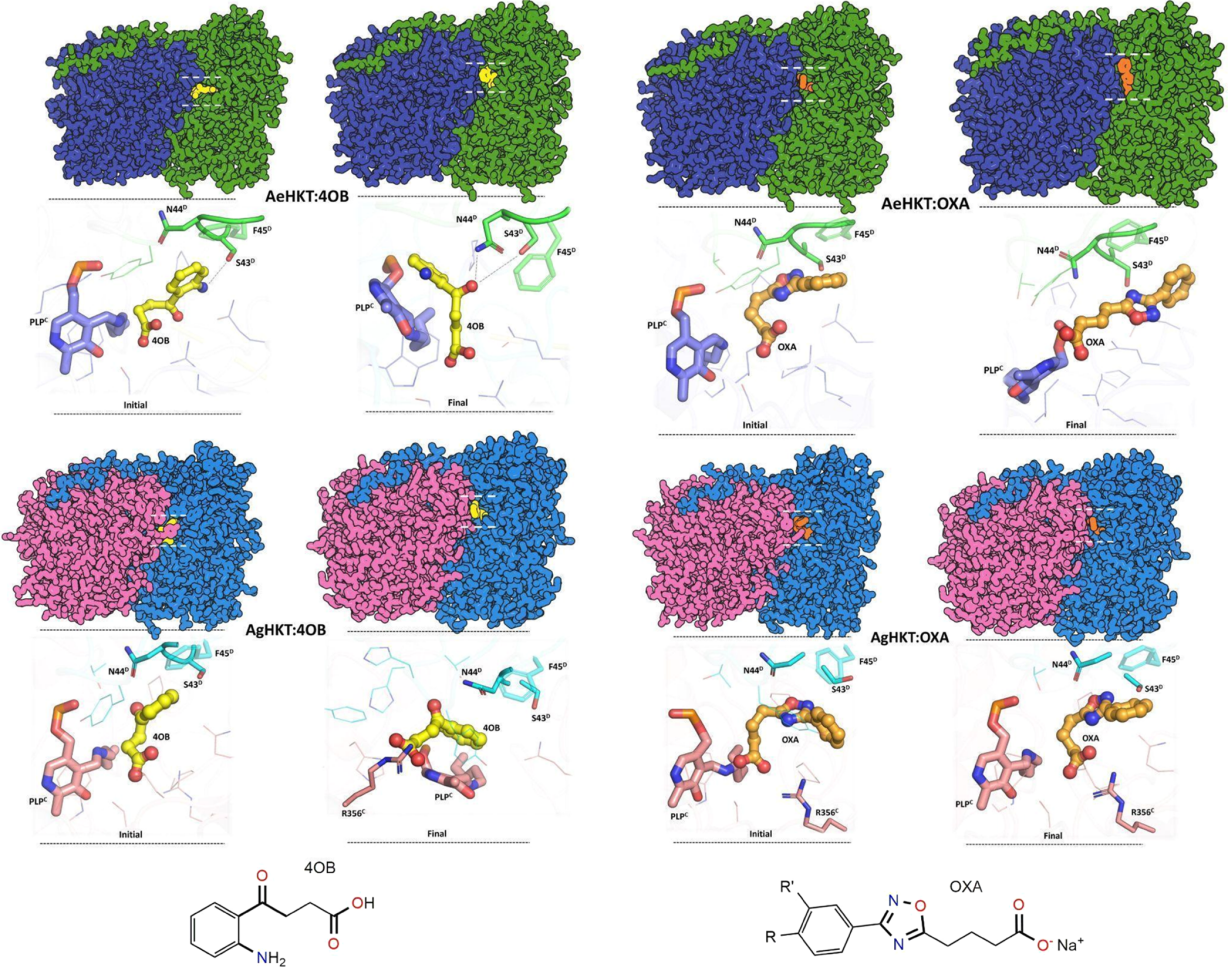
Initial and final conformations of four systems from MD simulations. AeHKT (green/blue) and AgHKT (pink/blue) bound to 4OB (yellow) and OXA (orange). The PLP cofactor and closest neighboring residues are also shown. Chemical structures of the inhibitors 4-(2-aminophenyl)-4-oxobutyric acid (4OB) and sodium 4-(3-phenyl-1,2,4-oxadiazol-5-yl)butanoate (OXA) are also shown.

The MD simulations of the four enzyme complexes showed only local conformational changes (Figure 3). Therefore, the ligand interaction with active site residues was not expected to undergo significant changes. The intermolecular interactions with key catalysis residues were maintained throughout the simulations (Figures 4 and S1). Comparison between the initial and final configurations after 100 ns of MD simulations supports a common pattern of interactions among the four enzyme complexes (Figure 4). The key residues described by Rossi and co-workers^27^ in the experimental AgHKT/4OB complex are S43, N44, and R356, which are also observed in simulations (Figure 4).

**Figure 5:**
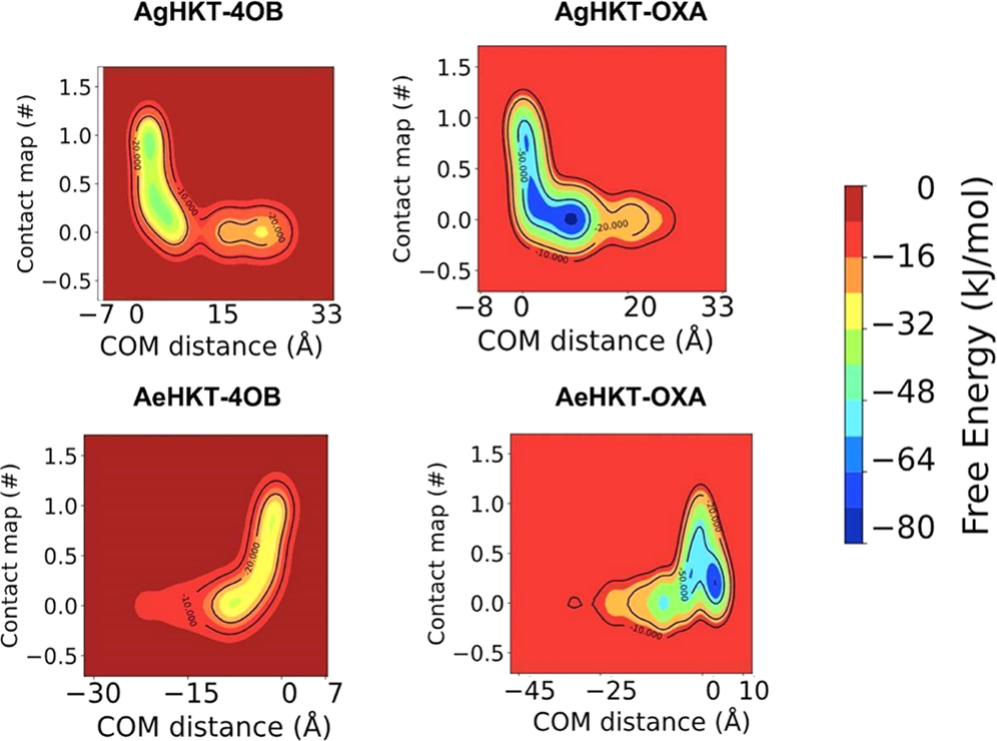
Three-dimensional free energy landscape for the dissociation of the ligands from the HKT enzymes as a function of the chosen CVs. The landscape is color-coded from red (high regions on the configurational space) to blue (low energy basins).

**Figure 6:**
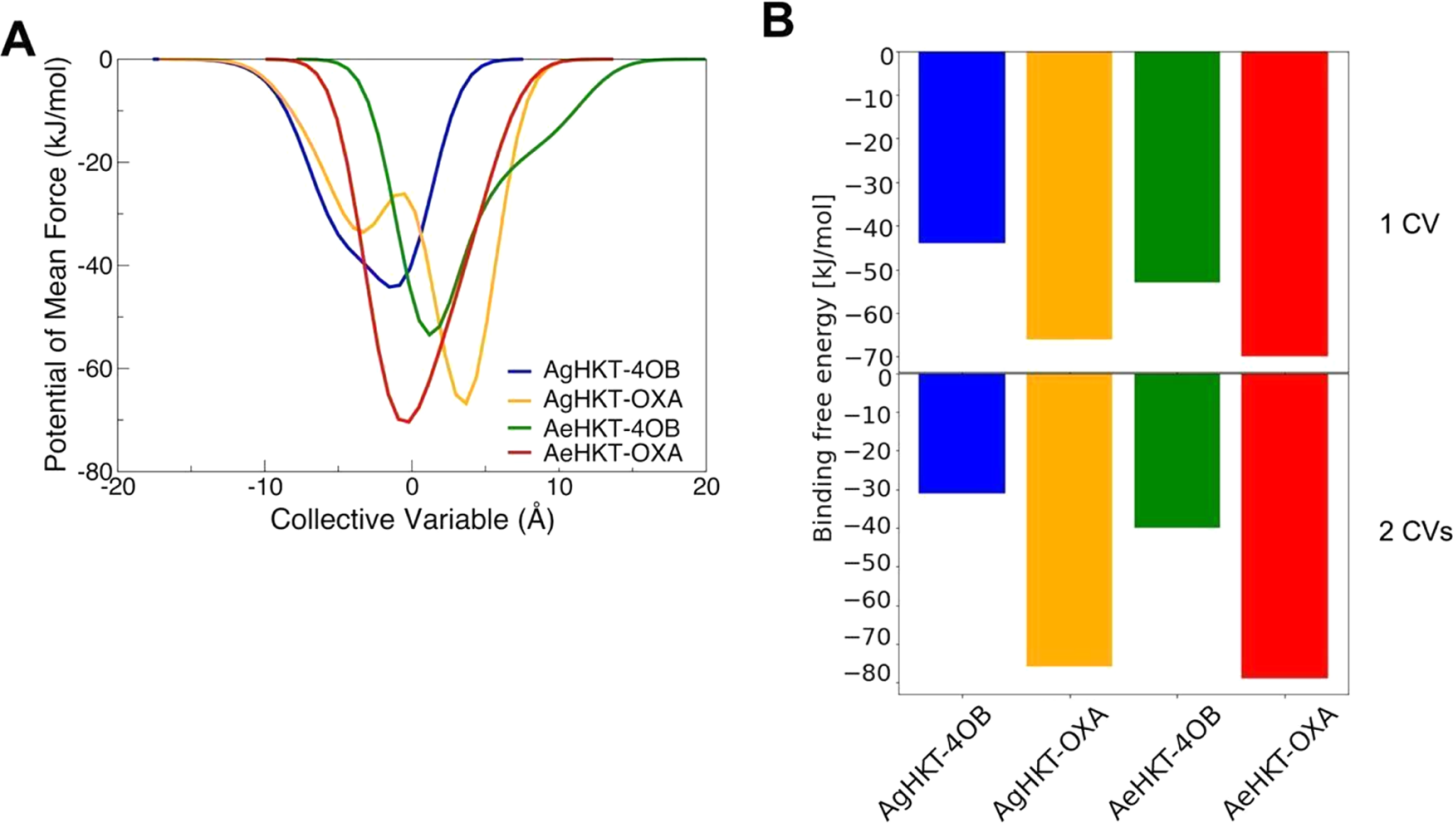
(A) Two-dimensional free energy surface landscapes for the dissociation of the ligands from the HKT enzymes depicted through the potential of mean force as a function of CV1. (B) Binding free energies calculated by meta-MD simulations, inferred from the free energy landscape for the dissociation of the ligands from the active site of the HKT enzymes. The latter values are shown for the dissociation of the ligands using one (top) or two (bottom) CV for the dissociation of the respective ligands.

We applied enhanced sampling in nonequilibrium simulations to characterize the kinetics and thermodynamics mechanism for ligand unbinding from HKT enzymes. Enhanced sampling simulations were carried out to simulate the egression routes of the ligands as the timescales of the dissociation phenomena far exceed what is achievable by conventional MD simulations. Metadynamics (Meta-MD) simulations enhance the conformational sampling by enforcing the system to leave its energy minima and cross free energy barriers to explore conformations associated with other stable basins unreachable via conventional MD.^35^ The addition of a history-dependent fictitious and repulsive potential allows for the reconstruction of the free energy surface (FES) of the ligand dissociation from the enzyme active site.^36^ This has been carried out via steering of the ligands OXA and 4OB from the enzyme active sites through a pathway defined by two collective variables, namely, the distance and the contact maps between the center-of-mass (COM) of the ligand and the COM of the α-carbon atoms of the residues in protein within 3 Å from the ligand (Figure 5). The values of the dissociation-free energy barrier can then be interpreted as relative binding free energies for the ligand–enzyme complexes (Figure 6). The free energy landscape for the unbinding process as a function of the CVs showed that OXA binds to AeHKT and AgHKT with a well-defined minimum and dissociates via multiple local minima (Figure 5). Alternately, 4OB binds to both enzymes with larger and shallower energy basins. Notably, upon 4OB binding to AgHKT (COM ≈ 0 nm), the energy basin expanded along the y-axis defined by the occupancy percentage of contacts between the ligand and enzyme atoms (Figure 5). Hence, 4OB binds via multiple, equally probable, states and smaller energetic barriers compared to the OXA ligand. The calculated FES indicates that 4OB binds to AeHKT and AgHKT with lower binding affinities than OXA (Figure 6).

The ligand unbinding mechanisms for the four complexes were further explored through simulations using a single CV, i.e., the distance between the COM of ligands and of α-carbon atoms of residues within 3 Å from the ligand. Expectedly, the binding energy values calculated with one or two CVs differ in magnitude as distinct Gaussian parameters were used for the two sets of Meta-MD simulations (Figure 6). However, the two sets of binding energies maintain the same ranking, evidencing convergence of the Meta-MD simulations. Considering a single CV, the free energy surface for the unbinding of OXA from AeHKT exhibited an intermediate state prior to dissociation, characterized by a local minimum along the FES (Figure 5). This intermediate state is further associated with an additional barrier transition along the egress path, and therefore, the presence of a metastable state along the egress pathway of the ligand is an indicative of slower dissociation.^37^ It has been shown that slow dissociation plays a leading role in the duration of drug action.^38,39^ Therefore, we have investigated the dissociation kinetics of OXA/4OB from the enzymes AeHKT and AgHKT.

**Figure 7:**
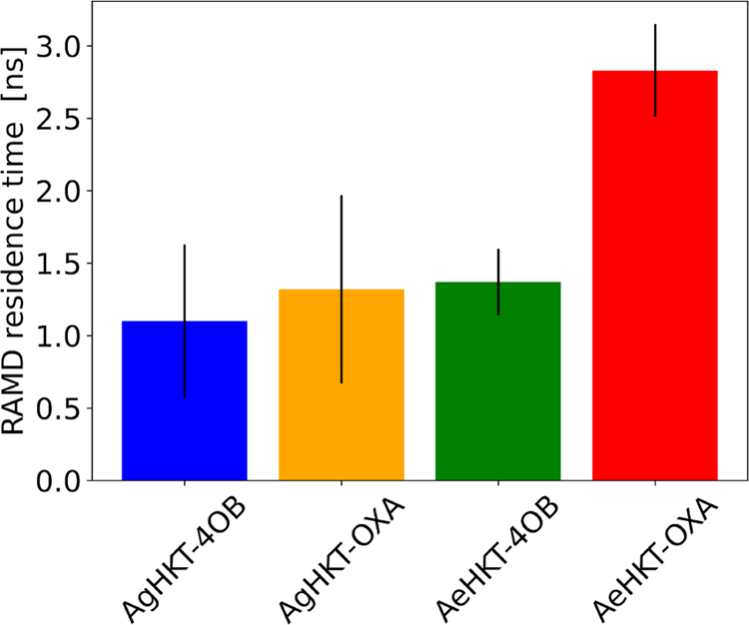
Relative residence time between the ligands and the HKT enzymes averaged over 6 τRAMD simulation replicas applying a random force of 20 kJ mol^–1^ Å^–1^ magnitude. The values of *τ* were averaged over six replicas, each one with 15 trajectories. The statistics to assess the convergence for each replica are shown in Figures S2–S5 from the electronic supplemental information (ESI).

The unbinding kinetics of ligand–protein complexes, given as the residence time τ and defined as 1/*k*_off_, has been shown to correlate better with the ligand binding efficiency than the binding affinity.^40−42^ The residence time can be related to the lifetime of the binary complex and is greatly influenced by the conformational dynamism of the active site of the target protein.^43^ Despite its relevance, the prediction of τ by brute-force MD is challenging, in part due to the inability to sample the timescale involved, typically orders of magnitude longer than what is trackable by conventional MD approaches. In addition, to compute kinetics parameters, the sampling of intermediate transition states between the bound and unbound states is also required. Therefore, to compute the τ for our systems, we have used a consistent approach that provided the relative τ for protein–ligand systems in the nanosecond timescale, referred to as τRAMD.^42^ The τRAMD protocol consisted of generating a large number of RAMD trajectories to dissociate the ligand from the protein active site through the application of a random force. It was applied to calculate the relative τ for the four complexes between OXA/4OB and the HKT enzymes (Figure 7). Except for AeHKT-OXA, all of the complexes showed equivalent residence times, which indicated similar *k*_off_. However, AeHKT-OXA exhibited a 2-fold increase in the residence time τ, implying enhanced binding properties (Figure 7). These findings are consistent with the Meta-MD simulations utilizing only one CV for which one intermediate state is present (Figure 5), therefore establishing an extra step before full dissociation. This behavior, observed only for the AeHKT-OXA system, is supposedly related to the opening–closing motion of the loop comprising A-Gln^253^-A-Arg^255^ residues since this is the major dynamical difference between the four systems.

As requested by one reviewer, we performed end-point calculation using the molecular mechanics Poisson–Boltzmann surface area (MM-PBSA)^44^ (Table S1). The free energy of binding between the ligands HKT enzymes was estimated for all four complexes. The free energy terms were decomposed into the individual contributions to the binding energy (Table S1). Overall, the free energy methods obtained via the MM-PBSA approach followed the same trend obtained from the metadynamics and τRAMD simulations. The single exception was AeHKT-4OB, but the magnitude of the errors associated with them, PBSA estimated free energies (Table S1), makes these calculations too qualitative. It has been extensively shown that even though MM-PBSA is computationally efficient and nowadays easily accessible for usage in all major MD software, the errors associated with this approach are very large (for a review, see refs (45, 46)). Most importantly, this technique has a much lower accuracy than enhanced sampling methods such as metadynamics. Among some pitfalls that could explain the discrepancy between the MM-PBSA and the metadynamics method employed in this work are the use of implicit solvation and the lack of conformational entropy by the former. These two components are critical for the appropriate description of protein–ligand recognition and binding. In any case, the MM-PBSA calculations corroborate the initial hypothesis that OXA ligands can be as (or more) efficient as (than) 4OB.

## Conclusions

The populational control of *Aedes* spp. is key toward the mitigation of arboviral diseases. Toward this end, we have determined the X-ray structure of the enzyme hydroxykynurenine transaminase (AeHKT) as a promising molecular target for the discovery and development of larvicide candidates against the *A. aegypti* mosquito. The HKT family of enzymes is specific to mosquitoes and has less than 20% sequence identity with its human cognate kynurenine aminotransferases I (KAT-I) and II (KAT-II),^25,26^ which makes them an unlike target for HKT-selective inhibitors. To date, the only available X-ray structure of HKT is from the malaria vector *A. gambiae* (AgHKT).^27^ Previously, it was shown that AeHKT is inhibited by 1,2,4-oxadiazole derivatives with IC_50_ values ranging from 35 to 340 μM via a noncompetitive mechanism.^17,18^ In this work, we have investigated the binding free energies and dissociation kinetics of the complexes between the inhibitors 4-(2-aminophenyl)-4-oxobutyric acid (4OB) and sodium 4-(3-phenyl-1,2,4-oxadiazol-5-yl)butanoate (OXA) and the enzymes AeHKT and AgHKT. We have found that OXA binds to both AeHKT and AgHKT enzymes with binding energies 2-fold more favorable than the crystallographic inhibitor 4OB. The inhibitor OXA also displays a 2-fold greater residence time τ upon binding to AeHKT compared to 4OB. In conclusion, the determination of the X-ray structure of HKT from *A. aegypti* broadens the small repertoire of mosquito proteins available for the design and discovery of selective inhibitors. It also paves the way for the further optimization of 1,2,4-oxadiazole derivatives as the larvicide candidates with improved affinity and selectivity.

## Materials and Methods

### Enzyme Expression and Purification

All reagents when not specified were purchased from Sigma-Aldrich. We have previously reported the cloning methods to obtain the recombinant HKT expressed in *E. coli* and the same construct was used here to perform crystallization trials.^17^ In this work, Rosetta 2 *DE3* cells containing AeHKT construct were incubated in ZYM-5052 autoinduction medium supplemented with 50 μg mL^–1^ kanamycin and 34 μg mL^–1^ chloramphenicol at 37 °C and 200 rpm until an OD_600_ of 0.6 was reached. Then, the temperature and rotation were decreased to 18 °C and 180 rpm for 32 h to induce protein expression. The cells were harvested by centrifugation at 4000*g* for 20 min at 4 °C. The pellets were suspended in lysis buffer (20 mM Tris pH 8, 200 mM NaCl, 10% glycerol (m/v), 10 mM imidazole pH 8, and 1% Tween 20 (v/v)), and 4 mM DTT, 1 mM phenylmethylsulfonyl fluoride, 10 U mL^–1^ benzonase nuclease, and 1 mg mL^–1^ lysozyme were added to the pellet solution and incubated in ice for 30 min. After chemical lysis, the solution was submitted to mechanical lysis by sonication (8 pulses of 30 s with 45 s interval). The bacterial debris were removed from the protein extract by centrifugation at 18,000*g* for 40 min at 4 °C. The supernatants were filtered by a 0.45 μM PTFE membrane and loaded into a HisTrap HP 5 mL column (GE Healthcare). Equilibration, injection, and washing steps were performed in buffer A (50 mM Tris pH 8, 500 mM NaCl, 20 mM imidazole pH 8, and 10% (m/v) glycerol), and elution was set in a 20–500 mM imidazole gradient. The recombinant HKT was eluted in 60% buffer B (50 mM Tris pH 8, 500 mM NaCl, 500 mM imidazole pH 8 and 10% (m/v) glycerol). Then, buffer exchange chromatography was carried out in a HiTrap Desalting 5 mL column equilibrated with buffer D (50 mM Tris pH 8, 200 mM NaCl) to remove imidazole excess. The removal of 6xHis and Trx tags was performed by adding 10% (v/v) of 1 mg mL^–1^ TEV protease and 4 mM DTT to HKT solution. The mixture was incubated for 3 h at 25 °C. After incubation, second affinity chromatography was applied to remove TEV protease, 6× His and Trx tags, and any uncleaved protein. Final buffer exchange chromatography was set under the same conditions described earlier, and the purified protein was concentrated until 20 mg mL^–1^. The protein was quantified in several dilutions by NanoDrop using a theoretical extinction coefficient estimated by ProtParam (61,475 M^–1^ cm^–1^).^47^ All steps were monitored by 10% SDS-PAGE.

### Crystallization, Data Collection, and Structure Determination

Crystallization screening was first set in 96-well plates by a sitting drop vapor diffusion technique at 18 and 4 °C. The crystallization plates were constructed by adding 2 μL of 5 or 10 mg mL^–1^ HKT and equivalent reservoir solution equilibrated against 100 μL of reservoir solution. Plate-shaped crystals were observed in 5 mg mL^–1^ HKT with JBScreen Classic kit (Jena Bioscience) after 10 days of incubation at 4 °C in reservoir solution containing 10% (w/v) poly(ethylene glycol) 8000, 10% (w/v) ethylene glycol, and 100 mM HEPES pH 7.5. Crystal optimizations were performed in hanging drop plates containing 2 μL of HKT and equivalent reservoir solution equilibrated against 1000 μL of reservoir solution, and the crystals were observed after 9 days of incubation at 4 °C. Single crystals were quickly immersed in a cryoprotectant solution containing 20% (v/v) of ethylene glycol in reservoir solution and then flash frozen in nitrogen foam at 100 K. All data were collected at the MX2 beamline at the Brazilian Synchrotron Light Laboratory using a wavelength of 1.45861 Å and Pilatus 2M as a detector. The dataset was collected covering 360° of the crystal. Diffraction patterns suggested an orthorhombic crystal with the *P*2_1_2_1_2_1_ space group. Unit cell dimensions were calculated as *a* = 86.24, *b* = 115.03, *c* = 171.04 and α = 90.00, β = 90.00, γ = 90.00. The molecular weight of a monomer is 43.4 kDa, and the asymmetric unit was defined by two homodimers. Data collection statistics are reported in Table 1. Data processing was performed by indexing and integration with XDS^48^ and scaling with AIMLESS^49^ and POINTLESS.^50^ A total of 59,594 unique reflections were considered, and they showed completeness >99% at 2.5 Å resolution. Initial phases were obtained by molecular replacement in Phaser^51^ using the homologous *A. gambiae* 3-hydroxykynurenine transaminase (79% sequence identity, PDB ID 2CH1(27)) as a model. Refinement steps were performed automatically by Phenix.refine^52^ and improved by iterative model building with Coot.^53^ Residues between Lys^308^ and Arg^313^ were constructed manually using Coot. Five percent of reflections were put aside from refinement to compose the test set (*R*_free_). The quality of the final model was assessed by MolProbity,^54^ and three Ramachandran outliers were present, but the φ and Ψ angles of these residues in the model were consistent with their electronic density. The water molecules were added automatically by Phenix.refine and confirmed by evaluation of *F*_o_ – *F*_c_ (1σ) difference maps using Coot. The link between Lys^205^ and PLP in each chain was made by Refmac in the last refinement step and then, the model showed *R*_work_ and *R*_free_ values of 19% and 26%, respectively. The coordinates and structure factors of AeHKT were deposited in the Protein Data Bank with an accession code 6MFB. Structural alignment was made by Pymol^55^ and sequence alignment was first made by Clustal Omega,^56^ and the aligned sequences were submitted to ESPript server^31^ to generate Figure 2.

**Table 1:** Data Collection and Refinement Statistics^a^

	AeHKT
Data Collection	
space group	*P* 2_1_2_1_2_1_
observations	388,427 (30,462)
unique reflections	59,594 (4580)
resolution (Å)	47.85–2.50 (2.57–2.50)
*R*_meas_^b^	0.094 (0.866)
*R*_p.i.m._^c^	0.037 (0.333)
multiplicity	6.5 (6.7)
completeness (%)	99.9 (100.0)
*I*/σ_(I)_	16.2 (2.6)
CC_1/2_	0.998 (0.896)
cell dimensions	
*a*, *b*, *c* (Å)	86.24, 115.03, 171.04
α, β, γ (deg)	90.00, 90.00, 90.00
Refinement	
*R*_work_^d^	0.19
*R*_free_^d^	0.26
RMSD^e^ bond lengths (Å)	0.010
RMSD^e^ bond angles (deg)	1.443
average B-factor (Å^2^)	51.35
protein (Å^2^)	51.61
PLP (Å^2^)	49.01
water (Å^2^)	43.35
no. of atoms (non-H)	
protein	12,080
PLP	60
water	370

a Data in parenthesis corresponds to the highest resolution shell.

b .

c .

d .

e r.m.s.d, root-mean-square deviation.

## Computational Procedure

### System Setup and Force Field Parameters

The atomic coordinates for *A. gambiae* (AG) and *A. aegypti* (AA) HKT were retrieved from the PDB database (ID 2CH2(27) and 6MFB), respectively. The structures of the HKT enzymes complexed with the ligands 4OB and OXA were prepared using the lowest energy structure obtained from previously published docking calculations.^16^ The missing atoms in the residue Glu^145^ from the chain B of the PDB ID 2CH2 were modeled while keeping the coordinates for all of the other atoms constrained using the score_jd2 application from the Rosetta package of software.^57^ The systems were centered and explicitly solvated in an orthorhombic box with 20 Å from the edges. Counterions of sodium and chloride were added to neutralize the total charge of −9e for AeHKT (161 Na^+^ and 152 Cl^–^) and −8e for AgHKT (160 Na^+^ and 152 Cl^–^) and to reproduce a physiological saline concentration of 150 mM. The simulations were performed using the GROMACS 2019^58^ engine. The protein, counterions, and water were described by the parameters contained in GROMOS 54A7,^59^ GROMOS 53A6^60^ force fields, and single-point charge (SPC)^61^ water model, respectively. The topologies and parameters describing the bonded terms and van der Waals interactions for the ligands and the PLP cofactor were obtained using the Automated Topology Builder (ATB)^62^ webserver (https://atb.uq.edu.au/) compatible with the GROMOS 54A7 parameters set. The input structures of the ligands provided for the ATB server were geometry-optimized using the density functional theory approach with the Pople-style 6-31g(d,p) basis set under Becke’s three-parameter hybrid (B3) function combined with the Lee, Yang, and Parr’s (LYP) correlation functional (DFT/B3LYP). The optimization of the ligands’ geometry was conducted in the gas phase. The partial atomic charges of the ligands and PLP were computed from the electron density population at the HF/6-31G* theory level, followed by a restrained electrostatic potential (RESP) fitting. The quantum chemical calculations were performed using Gaussian 09 software.^63^ The PLP molecule was covalently bonded to Lys205 to form lysine-pyridoxal-5-phosphate. The p*K*_a_ of the PLP molecule is 1.8 due to numerous surrounding residues rich in hydrogen-bonding donors/acceptors; thus, both hydroxyls at the phosphate group were represented in the deprotonated state. A cutoff distance of 14 Å for nonbonded Coulombic and Lennard-Jones interactions was used. The particle-mesh Ewald^64,65^ treatment of long-range Coulombic interactions was used beyond a cutoff of 14 Å with a fourth-order interpolation of charges on a 1.6 Å Fourier spacing.

### Classical Atomistic Simulations

For all simulations, the systems were initially energy-minimized using 5000 steps of the steepest descent algorithm and then equilibrated in a stepwise fashion. Initially, the system was heated to 303.1 K in the NVT ensemble for 500 ps with 100 kJ mol^–1^ Å^–1^ harmonical position restraints on the heavy atoms of the solutes. Initially, the velocities were assigned by a Maxwell–Boltzmann distribution at 5 K. Then, equilibration was carried out in the NPT ensemble by releasing the restraints in 3 steps, each of 500 ps with force constants of 50, 10, 2 kJ mol^–1^ Å^–1^. Then, the production was carried out for 100 ns without positional restraints on the NPT ensemble. The temperature was kept constant at 300 K using a velocity rescaling scheme^66^ with the solute and the solvent coupled separately to heat baths with a coupling constant of 0.4 ps. The pressure was isotropically maintained at 1 bar using a weak coupling Berendsen barostat^66^ with a coupling constant of 0.4 ps and compressibility of 4.5 × 10^–5^ bar. The leapfrog algorithm^67^ was employed to integrate the equations of motion with a 2.0 fs time step with all covalent bonds involving hydrogen atoms constrained by the LINCS algorithm.^68^

### Metadynamics Simulations

Enhanced sampling MD simulations were employed using metadynamics (Meta-MD) simulations^35,69^ by adding history-dependent Gaussian-like potentials to the Hamiltonian of the system. The simulations were carried out based on the last MD snapshot, using the same setup as described previously, except for the barostat, in which the Parrinello-Rahman (PR)^70,71^ scheme was used with a relaxation time of 2.0 ps. The PR pressure coupling was employed since it provides the exact fluctuation of the NPT ensemble and therefore yields thermodynamics quantities more consistently. To accelerate the sampling and to reconstruct free energy landscapes, two collective variables (CVs) were used to steer the ligands from the binding site and gain insight into the binding thermodynamics. The choice of the CVs was reasoned to ultimately describe the slow events relevant to describe the phenomena and to be able to distinguish transition states.^72^ Thus, the chosen CVs were CV1. The distance between the center-of-mass (COM) of the atoms of the ligands, and the COM of the α-carbons of the residues distancing 3.5 Å from the ligand CV2. The contact numbers derived from a switching function were calculated from the distance between the center-of-mass (COM) of the atoms of the ligands and the COM of the α-carbons of the residues. The form of the switching function is given as (eq 1)
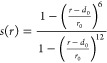
1in which *r* is the defined distance, *d*_0_ = 0, and *r*_0_ = 1. The parameters to compute the Gaussian potential were estimated based on the suggestions of Laio et al:^73^ the Gaussian width was set as 0.2 nm, a Gaussian weight of 0.25 kcal mol^–1^, and these were inserted at each 2 ps. In addition, metadynamics simulations utilizing only CV1 were performed using the same Gaussian potential parameters.

### τ-Random Acceleration Molecular Dynamics (τ-RAMD) Simulations

To compute the relative residence time (τ) of the protein–ligand systems, the τ-RAMD protocol^43^ was used. In τ-RAMD, the relative τ is calculated based on the simulation time that the ligands are dissociated from the active site through the application of a randomly oriented force. The force is applied to the center-of-mass of the ligand if its direction is altered during the RAMD simulation according to the pulling distance of the ligand. For each system, six replicas of 4 ns equilibration by conventional MD simulations were generated starting from the structure corresponding to the lowest point of the reconstructed FES by Meta-MD simulations. The equilibration followed the same setup as for the Meta-MD simulations; however, the Nose–Hoover thermostat was used with a relaxation constant of 1 ps. The last snapshot of each replica was used to initiate the dissociation by τ-RAMD simulations. For each replica, 15 dissociation simulations were carried out, yielding 90 simulations for each system. A force with random direction and magnitude of 20 kJ mol^–1^ Å^–1^ was applied to the COM of the ligands, and the direction of the force was changed every 100 fs only if the ligand COM did not move away a distance greater than 0.025 Å. The criterion to stop the simulations was when the distance between the COM of the ligand and the protein was greater than 50 Å. The computation of the relative τ as a function of the simulation dissociation times is thoroughly described in ref (43).

## Supporting Information Available

MM-PBSA energy estimates; hydrogen-bonding analyses; and τRAMD statistics (PDF)
