# Supplementary material for: Inhibition of 3-Hydroxykynurenine Transaminase from Aedes aegypti and Anopheles gambiae: A Mosquito-Specific Target to Combat the Transmission of Arboviruses
Source: ACS Bio Med Chem Au. 2023 Feb 16;3(2):211–22. doi: 10.1021/acsbiomedchemau.2c00080 (PMC10125267; doi:10.1021/acsbiomedchemau.2c00080)
Supplement: Supplementary file 1 — bg2c00080_si_001.pdf [file bg2c00080_si_001.pdf]

ELECTRONIC SUPPLEMENTAL INFORMATION

Inhibition of 3-Hydroxykynurenine Transaminase from *Aedes aegypti* and *Anopheles gambiae*: A Mosquito Specific Target to Combat the Transmission of Arboviruses.

Larissa G. Maciel[1,#], Matheus V. F. Ferraz[1,2,#], Andrew A. Oliveira[3], Roberto D. Lins[2], Janaína V. dos Anjos[1], Rafael V. C. Guido[3], Thereza A. Soares[4,5]**

[1]Department of Fundamental Chemistry, Federal University of Pernambuco, 50740-560 Recife, Brazil

[2]Aggeu Magalhães Institute, Oswaldo Cruz Foundation, 52740-465 Recife, Brazil

[3]São Carlos Institute of Physics, University of São Paulo, 13563-120 São Carlos, Brazil

[4]Department of Chemistry, University of São Paulo, 055508−090 Ribeirão Preto, Brazil

[5]Hylleraas Centre for Quantum Molecular Sciences, University of Oslo, 0315 Oslo, Norway

Corresponding authors: rvcguido@usp.br (RVCG)*;* thereza.soares@usp.br (TAS)

#These authors contributed equally to the work.

Table of Contents

1. Materials and Methods 2
1.1. Hydrogen Bond Analysis..2
1.2. Molecular Mechanics Poisson-Boltzmann Surface Area (MM-PBSA) 2
2. Supplementary figures (S1-S5)...3
3. Supplementary references 8

1. Materials and Methods

1.1. Hydrogen Bond Analysis

The number of hydrogen bonds between the ligands and surrounding residues within 6 Å of distance was computed along the simulation time. The GROMACS *gmx hbond* routine was employed. A 3 Å cutoff distance was used as hydrogen bond definition criteria for the donor-acceptor pairs.

1.2. Molecular Mechanics Poisson-Boltzmann Surface Area (MM-PBSA)

In addition to metadynamics, MM-PBSA calculations were carried out to estimate the binding free energy between the ligands OXA and 4OB from the HKT enzymes. The g_mmpbsa tool,[1] a GROMACS and APBS-based implementation of the MM-PBSA method, was used to perform the calculations. Snapshots were taken every 105 ps per complex for the last 50 ns of the production trajectories (resulting in a total of 460 frames per complex). The electrostatic contribution for the solvation free energy was estimated by solving the Poisson-Boltzmann equation,[2] whereas the nonelectrostatic term was taken into account through the SASA nonpolar model. For the APBS calculations,[3] the linearized form of the Poisson-Boltzmann equation was numerically solved using a grid spacing of 0.5 Å. The solvent was described by a dielectric constant of 80 and an ionic strength of 0.15 M NaCl, with atomic radii of 0.97 Å and 1.81 Å for the sodium and chlorine ions, respectively. The low-dielectric cavity was assumed as 4.

Table S1. Summary of the molecular mechanics Poisson-Boltzmann surface area (MM-PBSA) calculations. The estimated free-energies were decomposed and total binding free energy terms for the interaction between the ligands 4OB and OXA and the target enzymes, AeHKT and AgHKT.

System	van der Waal[a]	Electrostatic[a]	Polar solvation[a]	SASA[a]	Binding energy[a]
AeHKT-4OB	-87.70 ± 9.07	-41.71±17.70	104.10±20.76	-10.89±0.87	-38.20±9.82
AeHKT-OXA	-98.67±13.57	-46.92±18.03	111.94±25.36	-11.77±1.27	-45.43±18.42
AgHKT-4OB	-79.20±11.13	-45.47±14.85	109.27±19.16	-11.53±0.94	-26.94±11.72
AgHKT-OXA	-107.09±30.85	-24.61±16.85	99.30±31.82	-12.72±2.68	-45.13±23.13

[a]All energies values are presented in kJ/mol.

2. Supplementary figures (S1-S5)

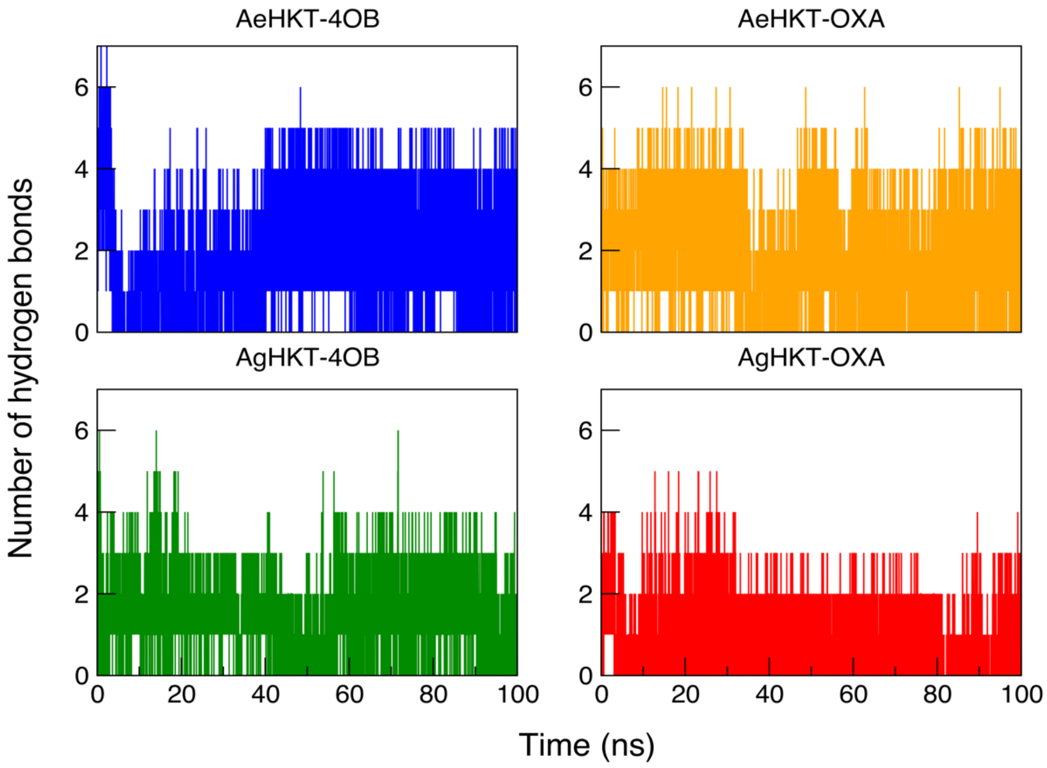

Figure S1. Time evolution series of the number of hydrogen bonds between the ligands and the surrounding residues within 6 Å of distance. The adopted criterion to be defined as a hydrogen bond was a distance cutoff of 3 Å between the donor and acceptor atoms.

τRAMD STATISTICS

Figure S2-S5. Statistics plots for the τRAMD protocol for computing the residence time for the ligands 4OB and OXA and the enzymes AgHKT and AeHKT. A total of six replicas were carried out and 15 dissociation trajectories were generated for each replica: (first raw) Cumulative distribution functions for the RAMD dissociation times of the six replicas; The effective residence time (for the raw data) is indicated as a red line, and it shows the simulation time at which dissociation was observed for 50% of the simulation runs. (second raw) Distribution function for the effective residence time post-bootstrapping (in blue). The Gaussian distribution is shown as a black line, and the mean residence time is shown in red lines. (third raw) Poisson cumulative distribution function shown in black line compared with the empirical cumulative density function in blue points. A red line shows the residence time for each replica and the Kolmogorov-Smirnov test is shown and calculated by the distance of the Poisson and empirical distribution functions. (fourth raw) Bar plots of the relative residence times averaged over each replica. The range of the data are shown by whiskers and the outliers are shown by points. Orange and dashed red lines denote the median and mean of the residence time, respectively.

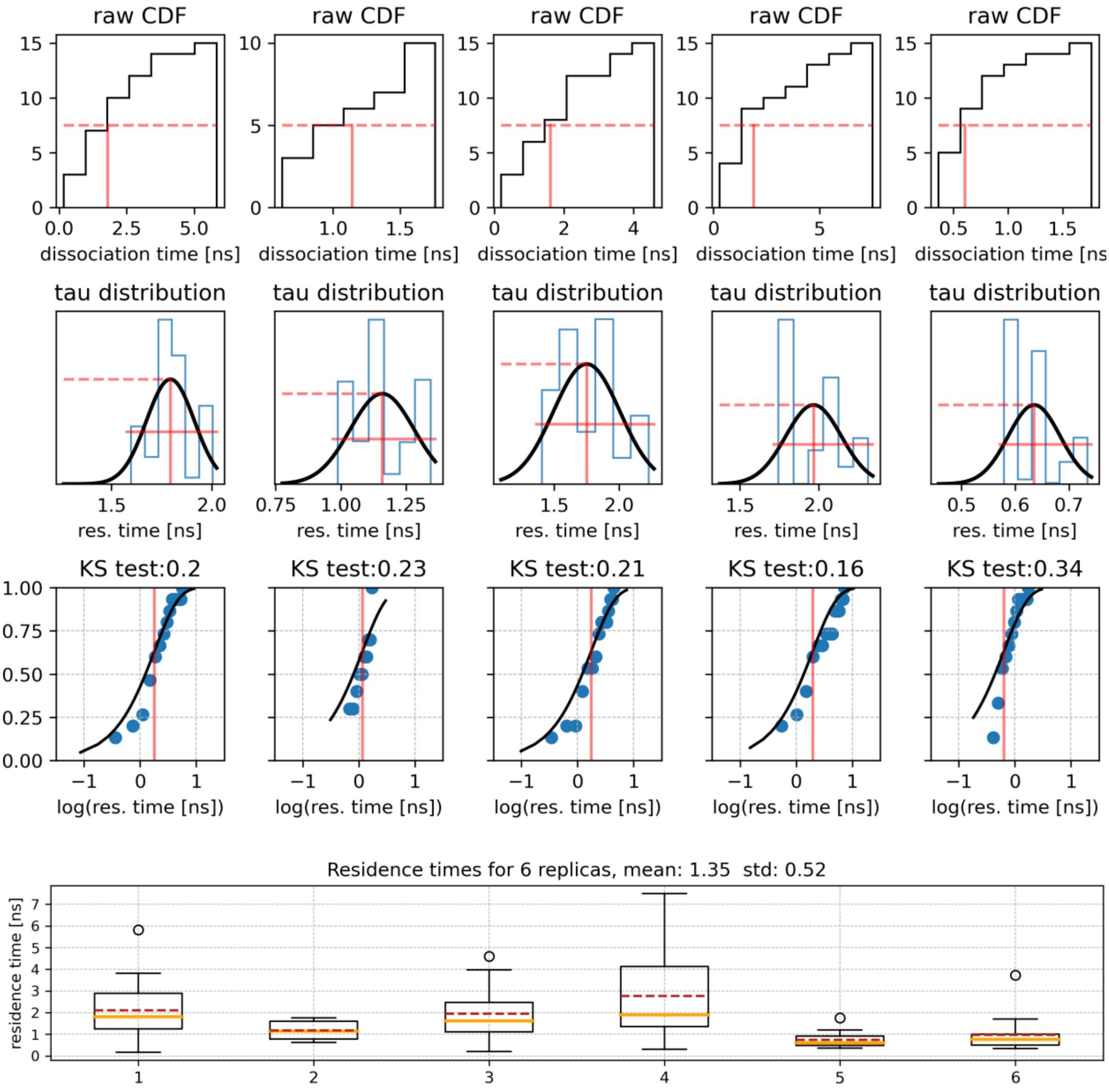

Figure S2. Statistics plots for the τRAMD protocol for computing the residence time for 4OB and the enzyme AeHKT (**AeHKT-4OB)**

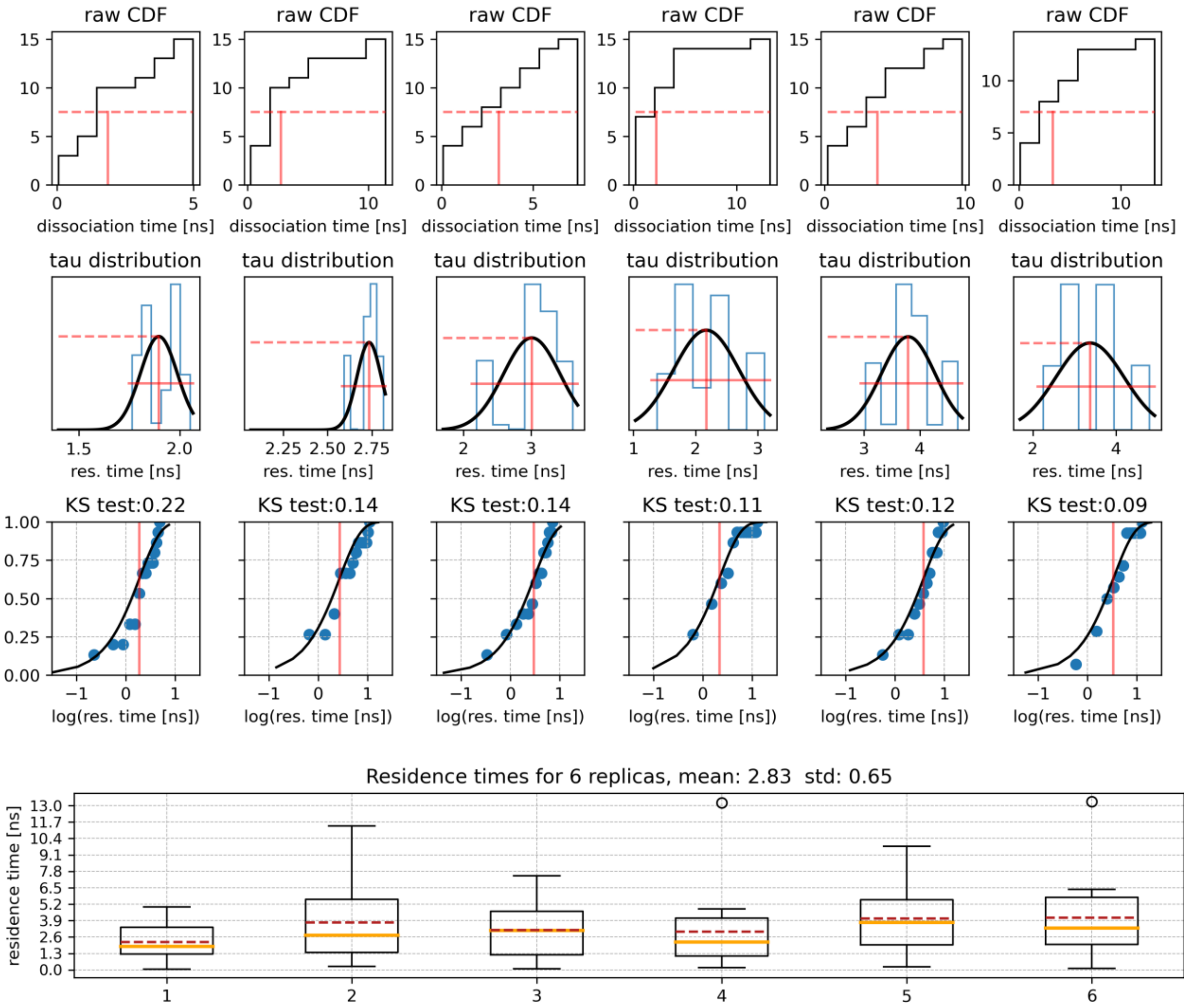

Figure S3. Statistics plots for the τRAMD protocol for computing the residence time for OXA and the enzymes AeHKT (AeHKT-OXA).

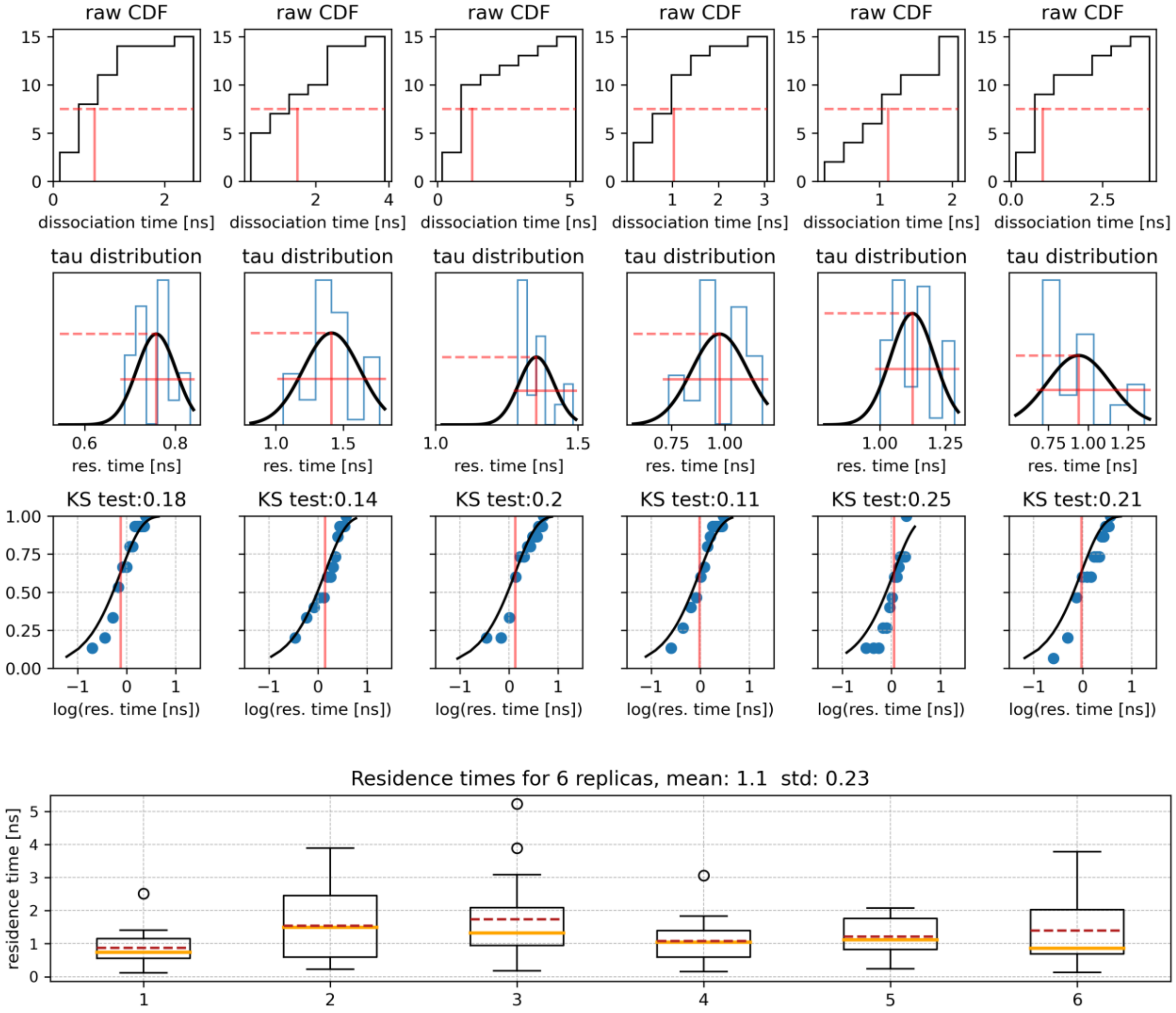

Figure S4. Statistics plots for the τRAMD protocol for computing the residence time for 4OB and OXA and the enzyme AgHKT (AgKT-4OB).

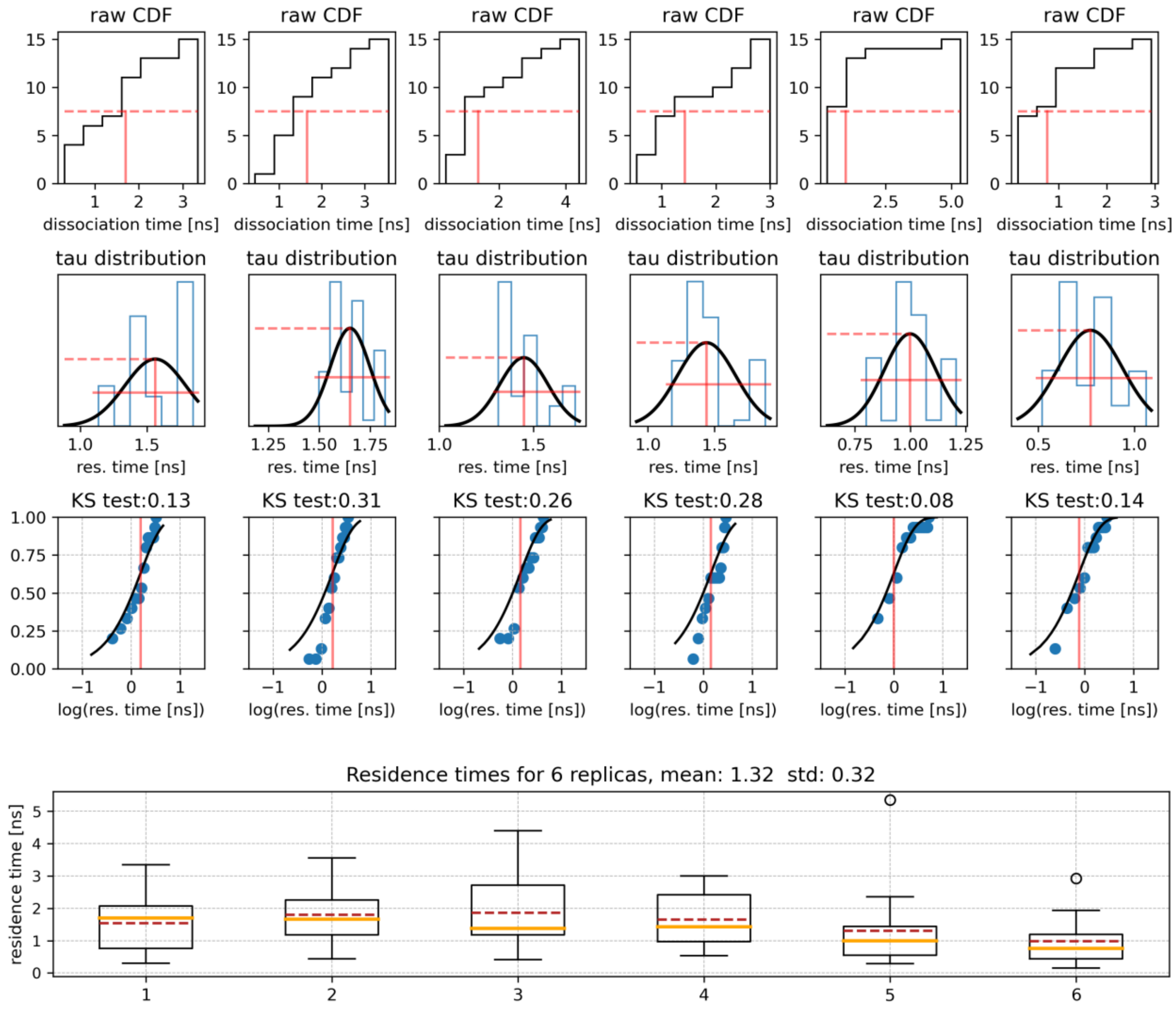

Figure S5. Statistics plots for the τRAMD protocol for computing the residence time for OXA and the enzyme AgHKT (AgHKT-OXA).

3. Supplementary references

(1) Kumari, R.; Kumar, R.; Lynn, A. G_mmpbsa —A GROMACS Tool for High-Throughput MM-PBSA Calculations. *J. Chem. Inf. Model.* **2014**, *54* (7), 1951–1962. https://doi.org/10.1021/ci500020m.

(2) Honig, B.; Nicholls, A. Classical Electrostatics in Biology and Chemistry. *Science (80-.).* **1995**, *268* (5214), 1144–1149. https://doi.org/10.1126/science.7761829.

(3) Baker, N. A.; Sept, D.; Joseph, S.; Holst, M. J.; McCammon, J. A. Electrostatics of Nanosystems: Application to Microtubules and the Ribosome. *Proc. Natl. Acad. Sci.* **2001**, *98* (18), 10037–10041. https://doi.org/10.1073/pnas.181342398.
